# Ethical and Legal Aspects of Technology-Assisted Care in Neurodegenerative Disease

**DOI:** 10.3390/jpm12061011

**Published:** 2022-06-20

**Authors:** Bjoern Schmitz-Luhn, Jennifer Chandler, on behalf of the iCARE-PD Consortium

**Affiliations:** 1 Center for Life Ethics, Bonn University, 53113 Bonn, Germany; 2 Bertram Loeb Research Chair, Centre for Health Law, Policy and Ethics, University of Ottawa, Ottawa, ON K1N 6N5, Canada; chandler@uottawa.ca

**Keywords:** ethics and law of medicine and technology, neurodegenerative diseases, technology-assisted care, liability, medical education

## Abstract

Technological solutions are increasingly seen as a way to respond to the demands of managing complex chronic conditions, especially neurodegenerative diseases such as Parkinson’s Disease. All of these new possibilities provide a variety of chances to improve the lives of affected persons and their families, friends, and caregivers. However, there are also a number of challenges that should be considered in order to safeguard the interests of affected persons. In this article, we discuss the ethical and legal considerations associated with the use of technology-assisted care in the context of neurodegenerative conditions.

## 1. Introduction

A plethora of promising technologies and applications is being considered for implementation in modern medicine and the health care sector. The field of technology-enabled care includes telemedicine, as well as various digital, electronic (eHealth), and mobile (mHealth) health services. These are increasingly being seen as a way to respond to the demands of managing complex chronic conditions [1]. A number of these emerging modalities are particularly useful in the context of neurodegenerative diseases such as Parkinson’s Disease, where they can help with the assessment and measurement of symptoms, monitoring of disease progression, supporting therapeutic decision making, facilitating rehabilitation and physical activity, and facilitating remote care [2]. For example, sensors can be used to closely monitor physiological functions to automatically detect changes in Parkinson’s-associated symptoms, monitoring the disease-related functioning and indicating any need for the adjustment of therapy, thus allowing patients to live more independently, e.g., in their own homes. In combination with algorithmic applications, the data collected from patients can be further used to predict the progression, duration, and outcome of the condition. The data can also be fed into a health-assisting and tracking platform, which, based on algorithmic calculations, can automatically alert physicians in case of symptom changes, sometimes even before these changes can be detected by the patient or in regular physical examinations.

Such systems can also be used to serve as a health platform for both patients and physicians, offering health advice to patients, letting them track their symptoms, facilitating symptom testing that would otherwise have to be performed in person, and overall reducing the need for the patient to regularly travel for examinations to the nearest specialized care center, which may, in fact, be located far away.

If individual patient data (including that collected via these care delivery technologies) are integrated into larger medical databases, machine learning techniques may be used to develop systems to support health care and medical research. The systems developed this way can aid medical professionals in assessing cases, predicting the course of the disease, and deciding on optimal treatment or prevention strategies. In medical research, they can help advance medical sciences by identifying patterns in large clinical and research databases, pointing to potential medically relevant causal relations to be further investigated by empirical and biological research. In turn, this research can, in some cases, be automated or simulated to speed medical discovery.

All of these possibilities provide a variety of chances to improve the lives of persons with Parkinson’s and their families, friends, and caregivers. However, there are also a number of challenges both from a legal and an ethical perspective that should be considered in order to safeguard the interests of affected persons. These will need to be addressed in order to contribute to the responsible use of technology in Parkinson’s care and ensure that its use is truly beneficial for patients and their caregivers.

The specific ethical and legal considerations that arise in relation to the use of technology-enabled care depend upon the particular technology, its role, and patient characteristics, among other things [3,4,5,6,7]. However, one of the central dimensions shared by the various techniques of telemedicine, mHealth, eHealth, and digital technologies is the collection, digital processing, transmission, and use of patient data. In this article, we discuss the ethical and legal considerations associated with the use of technology-assisted care in the context of neurodegenerative conditions such as Parkinson’s disease, focusing in particular on three phases of the collection and use of data: the collection of patient data (Section 2), the subsequent use of the collected data in providing care for individual patients (Section 3), and the secondary use of patient data within larger databases for medical research as well as insights through data-mining (Section 4).

## 2. The Collection of Data: Remote Diagnostics and Observation by Wearable and Ambient Sensors

The first area relates to the collection of data about disease symptoms, as well as environmental and social data, via sensors [8]. This can encompass, among many other applications, wearable sensors to measure changes in walking, tremor, or freezing of gait [9,10,11,12]. It can also include video monitoring on a dedicated computer on which regular tests can be administered [13] and general monitoring of the entire home to measure symptoms and, at the same time, ensure that the person is moving around safely.

Algorithms can be used to automatically assess the collected data on a real-time basis [14]. Deviations from what is typical for the individual in terms of symptoms and activity can be detected automatically and in real time. This allows for more rapid detection of changes in health and a possibility for informed professionals to intervene promptly. All of this could bring significant health benefits to those affected. On the other hand, such ambient assisted living technologies also bring up a number of ethical and legal considerations.

Some key questions are whether patients are sufficiently aware of the observational technology and the associated possible encroachments on their privacy, to what extent they have the opportunity to refuse or disable the observation, and whether their interests can be permanently protected given the potential for unanticipated secondary uses or the malicious misuse of recorded data.

The use of these technologies as part of treatment must meet the usual requirements for consent to medical treatment. In many jurisdictions, there is a legal requirement that voluntary and informed consent to treatment must be given by a capable patient or by a surrogate decision-maker if the patient is incapable. In addition, many jurisdictions also have laws requiring informed consent to the use of personal information. The need for informed consent in the context of digital medicine has been articulated at the international level [15]. In the context of neurodegenerative disorders, capacity, in particular, can be uncertain depending on the progression of the disorder [16]. Typically, a person is regarded as capable if the person can understand the information about the proposed treatment and can appreciate its implications for his or her own body and future life [17]. Particular difficulties arise from the fact that understanding the mechanisms underlying monitoring requires high levels of health and digital literacy on the part of those affected [18,19]. Understanding new technologies presents significant difficulties for many people—this is particularly true for people with cognitive impairments. If the patient lacks capacity, consent to monitoring and treatment must be first sought from a substitute or surrogate decision-maker. Legal systems vary in the mechanisms by which this person is identified, as well as the principles according to which they must give or withhold consent. Depending upon the jurisdiction, the options may include a guardian appointed by a court, a person previously formally selected by the patient (e.g., through a power of attorney), or a person specified by a statute (e.g., a family member or public guardian).

Patients may lose awareness over time of the potentially permanent collection of data, and its ongoing automated analysis as sensors are integrated into everyday life and surrounding objects [20]. Indeed, technologies with the capacity to sense and collect personal data are already increasingly being absorbed into the everyday environment [18]. The ability to make situational decisions about the desired degree of privacy can be significantly impaired as a result. Accordingly, an interesting legal question is whether and how a capable patient can practically revoke his or her consent to use the technology—while at the same time diminishing any dangers arising from sudden interruptions of the ongoing observation. This implies at least that sensor technologies should include clear and accessible ways for the patient to turn off or take off the device if they wish to do so voluntarily and that this should be distinguishable from accidental interruption or deactivation.

Data from wearable devices such as Fitbits have been used in various legal contexts, including murder trials [21]. An intriguing American case illustrates the questions that may arise with the use of medical devices that capture and record personal data. In Ohio v. Compton, the pacemaker data of a man accused of arson and insurance fraud were used in his trial to suggest his account of his activities was untruthful. The more continuous, comprehensive, and permanent the storage of data collected for medical purposes, the more useful it is for secondary purposes [21]. This must not only be considered in engineering these technologies (e.g., what is preserved and for how long) but also in disclosing risks to patients and seeking their informed consent.

While technology-assisted care can certainly enhance the quality of life by reducing the amount of necessary travel, especially in rural areas [22], the further development of sensor- and algorithm-based systems is likely to lead to a reduction in face-to-face human contact in care and nursing [23]. Instead of the expected improvement, the quality of life of some patients in need of care who have relied on regular nursing and examination visits may, in the end, be conversely reduced if these structures in the organization of their care were suddenly lacking. The ability of some patients to use complicated devices or platforms is also a question, and some may find it difficult to use these properly. This may affect the quality of care that can be offered to those patients, particularly if health care delivery is restructured on the assumption that these technologies will be used by all patients [24].

The issue of the financial accessibility of these new technologies can be a particular problem if technology-assisted care turns out to be more expensive than traditionally established structures in the health care system or is offered in addition to traditional care pathways [25]. With an increasing number of people affected by neurodegenerative diseases, more will be in need of treatment and ongoing care. In many cases, they will also be interested in living in their own homes for as long as possible. If the distribution of these technologies were to be based solely on free market principles, costly systems could be available only to well-off or privately insured individuals, at least for the foreseeable future.

However, technology-assisted care could eventually also become less expensive compared with care and support provided solely and directly by human caregivers and clinicians. While technology could increasingly replace human proximity and care [26], society will have to decide who should have access to what kind of care and under what conditions.

Finally, society must also clarify how the far-reaching consequences of ever more omnipresent sensor technology and continuously analyzed activity patterns and personality profiles are to be weighed against the interests of individuals in freedom and privacy in society.

## 3. The Application of Data: Predicting Disease and Supporting Medical Decision Making

Another field of technology use relates to the application of collected data for the purpose of automated predictions, warnings, or clinical decision support for health care providers.

### 3.1. Safety and Quality of Technology

Systems that automatically collect data and constantly monitor the health status of patients may improve patient safety and increase the efficiency of specialist medical activities by raising warnings in the case of changes in symptoms, offering curated disease history data and symptom prediction, and even suggesting therapeutic options to clinical staff [8,10]. Faster and more comprehensive access to relevant information allows treating physicians to make better decisions in a timely manner. In addition, support systems can contribute to significant improvements in prognostication by bringing together systematically organized information from the patient’s medical record and the latest medical publications and by automatically identifying relevant patterns [27,28]. Moreover, if the time a physician spends gathering information is reduced, time could ideally be freed up for other medical tasks, such as more detailed patient consultations and caring for more patients. Other benefits are that human errors caused by the limitations in the information or fatigue-related lack of focus may be reduced.

At the same time, it is not certain that medical decision-support systems will actually increase medical safety in all areas of application, and this must be explored and ensured. For example, the systems may be poorly designed and unreliable in their collection of input data, analytical algorithms, and communication of results. Should such systems achieve worse results than human decision makers in some areas, their use would be ethically and legally problematic. From a legal point of view, the most important factor is whether the decision-support technology meets the quality standards set by the general legal requirements for medical practice. Ultimately, it will be a question of whether the system contributes to decisions that meet the quality expectations of a conscientious specialist with the same training, i.e., whether the medical standard is maintained or undercut by the use of the system. The converse risk is that if healthcare providers do not adopt technologies that are shown to lead to a superior standard of care and have been accepted in the field as the appropriate mode of practice, this too could be seen as a failure to meet the shifting standard of care.

With the increasing collection and mining of personal health data, there is the possibility of more precise personalized medicine. Rather than elaborating treatment standards and guidelines for large groups, it is increasingly possible to have approaches suitable for smaller and smaller subgroups of patients. This also complicates the identification of the applicable standard of care for a specific patient, as the quality of care owed is increasingly individual rather than common across a larger patient group sharing a particular disease as a medical entity. In turn, it puts heavy demands upon health care providers who would be hard-pressed to keep track of the proliferating and more granular standards without relying upon expert systems. This can ultimately lead to a fragmentation of medical standards, a loss of clear rules of conduct in the doctor–patient relationship, and legal uncertainty.

In a way, recommendations generated automatically by decision-support systems, if based on correct and comprehensive data, can resemble medical guidelines. These guidelines also articulate an existing expectation of the general quality of medical care since they are also decision-making aids that have been systematically developed for specific diagnostic and therapeutic problems by specialist groups of physicians, albeit intended for application in a large number of cases. They, too, leave the physician a margin of decision and a corridor for action: guidelines may, must, or in some cases, must not be followed. The decisive factor is always the medical plausibility of the reasons for deviation in the specific treatment situation. Accordingly, physicians may tend to always follow the recommendations of algorithms even against their better judgment because of the concern about possible liability for their inadequately justified disregard of recommendations [29]. As with medical guidelines, following a recommendation resulting in harm may be more easily justified than deviating from the algorithm’s recommendations. This specifically holds true as algorithmic systems become increasingly capable of not only recommending treatment options but also anticipating the further course of the disease based on such a large amount of data that the algorithmic prognosis might not even be reproducible or explainable by a single human.

There are risks of undue deference by clinicians to complex algorithmic decision-making aids. These new types of predictive methods are often associated with uncertainty, especially in the case of so-called “black box” algorithms, where various methods of machine learning are used to produce a prediction that is opaque in the sense that users cannot comprehend, reconstruct, or verify the outcome of the calculation [30,31]. Such methods offer probability-bounded predictions rather than certainties, and false negative or false positive findings and projections are possible. Another danger lies in the fact that decision-support systems based on data mining cannot distinguish between correlations and causalities [32,33]. There is thus a risk of error if users put undue trust and an exaggerated reliance on systems that purport to draw causal inferences from patterns identified through the automatic analysis of large datasets. Human researchers can recognize such relationships because of their capacity to create hypotheses and their grounding in other areas of knowledge—an algorithm cannot do so.

### 3.2. Responsibility for Quality of Care

It is therefore important to clarify the conditions under which physicians should be allowed to ignore the recommendations of these types of decision-support systems. For example, machine-generated recommendations could be made expressly nonbinding for the treating physician depending on what risks are associated with a given intervention, what knowledge exists of its complication rate, and the known error rate of the recommendation system [29].

In the end, if no clear standard for the required actions of a physician exists (yet) in relation to how to use a specific decision-support system, the question of whether a physician’s reliance upon or disregard of its recommendations constituted an unreasonable error is hard to answer. Ultimately, the reasonableness of using or not using the system in the first place will turn on its availability and what is known about its strengths and weaknesses for a particular application. If it is reasonable to use it in general, a clinician must still bring to bear reasonable judgment on whether to follow or disregard the recommendations of the system in individual cases. This, too, will be affected by what is known about the functioning of the system and its error rates in particular types of cases and the plausibility and appropriateness of the recommendation for the particular patient.

The chain of accountability for poor decisions will be complicated, however, since many parties are involved, including the designers and developers of decision-support systems, the regulators who approve them, the hospital and clinic administrators who adopt them, and the health care providers that eventually use them in treating patients. In addition to the design of the underlying algorithms, the system’s performance also depends on many other factors, such as the quality of the data and correct handling by users. One can also imagine the damage that originates not in the data inputs or the design of the algorithms used for data analysis but from other sources. For example, data collection and transfer systems may be generally excellent for their intended purpose of supporting medical decision making while at the same time having poor cybersecurity, perhaps allowing malicious targeting of elderly people by accessing information about their routine behavior [34,35].

Accordingly, clear attribution of responsibility in the event of failures is difficult, and the diffusion of responsibility may undermine incentives to improve systems. Legal systems have not come up with a final solution; the identification of culpabilities of different actors and the exclusive or collective attribution of liability to them will continue to be part of policy and legal rulemaking. The application of existing rules to entirely new facts and circumstances is complex and, in many cases, uncertain as courts and jurisprudence are slowly but increasingly confronted with these issues.

### 3.3. Informed Consent

Another major challenge for the correct use of data and the use of algorithmic recommendations is the informed consent of the patient. Usually, treatment, as well as the collection of personal data, requires the patient’s consent. Its ethical foundation is respect for the autonomy of the person. The patient’s right to self-determination gives the fundamental right to decide freely to accept or reject treatment [36] and to base this decision upon the disclosure of risks, benefits, and alternatives by the practitioner sharing his or her pertinent medical expertise. In the case of algorithmic decision-support systems, it largely remains unclear what this means regarding the scope of the information needed for the patient to freely decide whether to consent to the use of such a system [37]. Indeed, simpler guidelines and checklists are routinely used in medical decision making, and this is not always discussed in detail with the patient, although elements of it may be discussed when explaining a physician’s recommended treatment options to the patient. When it comes to complex algorithmic decision-support systems, the question arises as to whether patients should be included in deciding upon their use in the patients’ own cases [38]. Risk disclosure in this context should include known safety or reliability issues of the technology, but the precise extent of necessary information regarding its functioning or its performance in comparison with a human physician remains opaque.

### 3.4. Medical Education

The increasing technological complexity and specialization of diagnosis and therapy also lead to new demands regarding the education of medical and care staff. On the one hand, specialization seems to suggest that a team of experts from different disciplines will often be needed to communicate specific information to patients. At the same time, the information needs of patients and their families and caregivers become increasingly wide, given the broad character of data used for algorithmic decision support and the wide implications for the everyday life of the patients and their caregivers as outlined above. In contrast to traditional care models, the management of disease becomes less of a clear medical decision about treatment and increasingly one about future planning, allowing monitoring technology into one’s private life, assessing both medical risks and benefits of the technology used as well as social implications of technology-assisted disease management. This transforms the role of the physician from a specialist medical guide into one who is at the center of technology, mediating between the technological and social aspects of care and funneling human compassion and needs between patient and the technology-assisted “medical world”.

In addition, the use of technologically complex tools increasingly requires medical staff to have a deeper understanding of the relevant technology, informatics, and statistics that reaches far beyond specialist medical knowledge in order not only to be able to relay these complex issues to the patient but also to correctly assess whether to trust the technology in a given situation or to know at what point to question it, to fulfill standards of care as outlined above [39,40].

In short, the arrival of digitization in care brings about fundamental changes in the professional requirements and new kinds of needed skills and competencies. Professionals will therefore need to be supported in their professional development in a way that optimally meets the needs of patients and the legitimate interests of caregivers.

### 3.5. The Therapeutic Relationship

The increasing use of technological systems for diagnosis, prognosis, and therapeutic decision making may also have an impact on the relationship between physician and patient [23,24,39,41]. They might strengthen that relationship by enabling the physician to devote more time to the individual patient both professionally and humanely [42], but they might also impair the relationship if physicians are reduced to “agents” and “translators” of recommendation systems and patients to “data subjects,” being regarded less as a person than as a “digital data stream” or exemplar of certain data characteristics. If undue deference to technological systems—“automation bias”—led to a loss of human and professional competence, this would not only increase the risk of incorrect medical decisions but also the risk of a fundamental loss of trust in the relationship between doctor and patient [23]. The human ability to respond to patients’ personal preferences, fears, and beliefs cannot be replaced by technology.

## 4. The Secondary Use of Patient Data: Building Digital Health Platforms and “Learning Health Care Systems”

The medical and other personal data collected by monitoring patients can be incorporated into databases, which can then be mined to advance medical knowledge about the risk factors associated with disease progression, comorbidities, etc.

Again, the comprehensive collection and automated analysis of patient data raise questions about the guarantee of informational self-determination and the protection of the data in question. In particular, there is the question of responsibility for the appropriate use of the data collected. Healthcare institutions are faced with the challenge of deciding who is allowed to collect and use what patient-related data for what purpose and over what period of time.

Complete anonymization of patient-related data is difficult to guarantee in practice [31,43]. This poses some potential risks for the individual from the disclosure of a current, past, or even prospective health status. Depending upon the nature of the information, it might also affect close relatives in their ”right not to know” if the information allows additional predictive insights to be drawn beyond the known neurodegenerative condition of their relative. To the extent that neurodegenerative diseases are inheritable, disadvantages regarding insurance or employment contracts in case of an adverse prognosis or incidental findings by the algorithm must also be taken into account [44].

Patients generally give their informed consent to process the collected data in the specific context of treatment or disease management, and consent is limited to these applications. If consent is given for further processing of data, e.g., for use in the context of clinical research, this release usually relates solely to use within the framework of the relevant clinical setting. By contrast, patients are usually unaware at the time of data collection of the possibility of digitally linking and evaluating data collected in a specific medical context, sometimes decades later and for purposes beyond the initial collection and use. It is rather strained to rely upon some form of “implicit consent” at the time of data collection for the open-ended future use of this data. Vast amounts of data that could be put to use for generating new medical insights, therefore, remain unused, while patients might actually have an interest in having them put to use. Furthermore, where data are being used, they are often pooled with exclusive access by a single large corporation, further decreasing the availability of data for medical research [45].

Solutions should be developed for improving data accessibility and ownership, fostering open source solutions, and enabling patients to consent to future uses of their data that are in their interest, e.g., by establishing models of meta consent and limiting corporate exclusivity [45]. On the other hand, medical institutions are required to develop suitable protection concepts and procedural rules to protect the interests of patients. The centralized aggregation of large amounts of data also generates appealing targets for unauthorized users of that data, making security requirements for the integrity of data platforms and protection from hackers or other unauthorized data access particularly important. In addition to the developers and the companies offering the corresponding algorithms, the medical institutions, as well as physicians and medical researchers themselves, should be provided with institutional regulations for dealing with the available data to ensure their responsible use and protection.

## 5. The Future: Responsible Use of Promising Technology

In order to practically realize the opportunities offered by advanced technology in health care delivery and to adequately meet the associated challenges, numerous measures are required at various levels.

A public discussion is due on how to deal with some of the implications brought about by the new technologies. Especially in publicly funded health care systems, societies will have to come up with preferences regarding resource allocation in light of promising but sometimes expensive new technological possibilities. If the investment is to be made, should health care systems prioritize digital disease prediction and prevention and technology-supported treatment for manifest diseases alone, and how will systems safeguard humane and empathetic care for those in need? This also includes dealing with the far-reaching consequences of ubiquitous sensor technology and continuous collection and analysis of activity patterns and derived personality profiles, all of which have potential implications for individual interests in freedom and privacy in society.

Medical professionals such as physicians and nurses must be prepared for and supported in the use of new technology-enabled care, which includes, in particular, teaching the necessary technical skills. Healthcare professionals must understand how algorithms work and how to interpret the results of algorithm-based decision-support systems. In designing predictive algorithms, especially in decision-support systems, it will often be necessary to deal with probabilities. Dealing with probabilistic risk statements is as challenging for many health care providers as it is for laypersons. In order for the systems to provide meaningful support, medical experts must be able not only to interpret the automatically generated results correctly but also to communicate this interpretation to patients in an appropriate and comprehensible manner.

It is also important to clarify how incidental findings should be handled. These are findings unrelated to the original objective of using the technology-enabled care method, but which are important for the person examined and possibly for his or her relatives. In the context of neurodegenerative conditions such as Parkinson’s, the data collected through sensors, mHealth applications and platforms, or telemedical examinations, when processed through various decision-support technologies, can identify additional risk factors or conditions. This may be significant for the patient, and to the extent that these risk factors or conditions are inheritable, it may also be significant for the patient’s relatives.

At the level of practice, there is an increased need for action to support the informed consent of patients and to protect their privacy. In the future, patient education may also have to include information about which algorithms are used in the course of diagnostic and therapeutic decision making and what risks and opportunities are associated with them. Currently, neither have the risks and opportunities been sufficiently clarified nor have adequate educational materials and approaches for their implementation been developed. Given the new possibilities in processing personal data, the specific practical requirements for obtaining informed consent also need to be clarified.

The trusting relationship between doctor and patient should be strengthened and secured, and institutional processes should be established to support the respective actors in making ethically sound and safe decisions in the best interest of patients.

Ethical aspects should be incorporated into the design and development process from the outset, for example, in interdisciplinary teams. It is also important that clear quality standards be developed for machine learning-derived algorithms to be used in health care decision making and a clarification of the shared responsibilities of developers, sellers, physicians, and other users in relation to the quality of the systems.

Technology has already proven to be a powerful and promising tool for many applications in medicine and care, greatly facilitating the work of health care providers and improving patients’ lives. In the end, the goal remains to enable and regulate a meaningful, safe, responsible, and beneficial collaboration between humans and machines. Such collaboration will be possible only if it is not solely oriented toward the technical possibilities but toward human needs.

## Data Availability

Not applicable.
